# Identifying Mechanisms by Which *Escherichia coli* O157:H7 Subverts Interferon-γ Mediated Signal Transducer and Activator of Transcription-1 Activation

**DOI:** 10.1371/journal.pone.0030145

**Published:** 2012-01-11

**Authors:** Nathan K. Ho, Ian Crandall, Philip M. Sherman

**Affiliations:** 1 Department of Laboratory Medicine and Pathobiology, University of Toronto, Toronto, Ontario, Canada; 2 Department of Pharmacy, University of Toronto, Toronto, Ontario, Canada; 3 Cell Biology Program, Research Institute, Hospital for Sick Children, Toronto, Ontario, Canada; National Jewish Health and University of Colorado School of Medicine, United States of America

## Abstract

Enterohemorrhagic *Escherichia coli* serotype O157:H7 is a food borne enteric bacterial pathogen that causes significant morbidity and mortality in both developing and industrialized nations. *E. coli* O157:H7 infection of host epithelial cells inhibits the interferon gamma pro-inflammatory signaling pathway, which is important for host defense against microbial pathogens, through the inhibition of Stat-1 tyrosine phosphorylation. The aim of this study was to determine which bacterial factors are involved in the inhibition of Stat-1 tyrosine phosphorylation. Human epithelial cells were challenged with either live bacteria or bacterial-derived culture supernatants, stimulated with interferon-gamma, and epithelial cell protein extracts were then analyzed by immunoblotting. The results show that Stat-1 tyrosine phosphorylation was inhibited by *E. coli* O157:H7 secreted proteins. Using sequential anion exchange and size exclusion chromatography, YodA was identified, but not confirmed to mediate subversion of the Stat-1 signaling pathway using isogenic mutants. We conclude that *E. coli* O157:H7 subverts Stat-1 tyrosine phosphorylation in response to interferon-gamma through a still as yet unidentified secreted bacterial protein.

## Introduction

Enterohemorrhagic *Escherichia coli* (EHEC), including the most common serotype O157:H7, is a non-invasive enteric bacterial pathogen that causes both sporadic cases and outbreaks of hemorrhagic colitis and hemolytic-uremic syndrome in humans [1]. Human zoonotic infections with EHEC occur through the ingestion of contaminated foodstuffs and water supplies, as well as from person-to-person transmission of the organism [2].

One of the first lines of host defense against bacterial insults is through activation of the innate and adaptive immune systems [3]. Pro-inflammatory cytokines, including interferon gamma (IFNγ), are secreted into the extracellular environment and activate an anti-microbial state in the body [4]. IFNγ production by macrophages, Natural Killer (NK) T cells and activated T cells triggers an antimicrobial state in host cells by binding to the IFNγ receptor, and tyrosine phosphorylation of the signal transducer and activator of transcription-1 (Stat-1) molecule. This activation leads to Stat-1 dimerization and translocation from the cytosol into the nucleus, where it binds to the gamma activating sequence (GAS) and triggers the up-regulation of up to 2,000 pro-inflammatory genes, including inducible nitric oxide synthase (iNOS), monocyte chemoattractant protein-1 (MCP-1) and lymphocyte adhesion protein ICAM-1 [5]. An intact IFNγ pathway is essential to combat infection initiated from a wide range of microbial pathogens; therefore patients with genetic defects in Stat-1 signaling are susceptible to microbial infections [6], [7], [8].

Subversion of the IFNγ/Stat-1 signal transduction pathway by microbial pathogens promotes bacterial colonization and prevents bacterial clearance from the host [3]. EHEC has evolved a method to subvert the IFNγ pathway, through a still unknown factor [9]. Therefore, the aim of this study was to determine how EHEC infection disrupts IFNγ signal transduction in human epithelial cells. The findings revealed that the IFNγ signal transduction pathway, important for host defense, is compromised at the level of Stat-1 tyrosine activation by an unknown EHEC secreted protein.

## Materials and Methods

### Tissue culture

HEp-2 epithelial cells (ATCC CCL-23) were used as a model epithelial cell line, as previously described [10]. Briefly, cells were grown in minimal essential medium (MEM) containing 15% (v/v) fetal bovine serum (FBS), 2% (v/v) sodium bicarbonate, 2.5% (v/v) penicillin streptomycin and 1% (v/v) amphotericin B (all from Invitrogen, Burlington, Ontario, Canada). Cells were grown in T75 flasks (Corning Inc., Corning, NY) at 37°C in 5% CO_2_ until confluent (8×10^6^ cells/flask). Confluent cells were trypsinized using 0.05% trypsin (Invitrogen) for 5 min at 37°C in 5% CO_2_. Trypsinized cells were then pelleted by centrifugation at 40g for 5 min (Beckman Coulter, Mississauga, ON, Canada), resuspended in MEM and re-seeded into either 6 well (Becton Dickinson Labware, NJ) or 24 well dishes (Corning Inc.) and grown at 37°C in 5% CO_2_ until confluent. Prior to bacterial infection, cells were incubated in MEM without antibiotics for 16h at 37°C in 5% CO_2_.

### Bacterial strains and growth conditions

Enterohemorrhagic *E. coli* O157:H7, strain EDL933 (EHEC) (accession: AE005174.2) and enteropathogenic *E. coli* O127:H6 strain E2348/69 (EPEC) (accession: NC_011601.1) were used in this study. Strains were cultured on 5% sheep blood agar plates (Becton, Dickinson and Company, Sparks, MD) at 37°C for 16h and stored at 4°C until use. Prior to infecting epithelial cells, bacteria were grown in 10 ml of static, non-aerated Penassay broth (Becton, Dickinson Co.) overnight at 37°C.

### Bacterial culture supernatants

To collect bacteria culture supernatants, ∼1×10^9^ CFU/ml of EHEC O157:H7 culture was centrifuged (3,000g, 15 min) and resuspended in 10 ml of serum free MEM without antibiotics. After growth for 24h at 37°C in 5% CO_2_, the medium was centrifuged (3,000g, 15 min), filtered (0.22 µm) and stored at 4°C. Sterility was confirmed by lack of bacterial growth of 0.1 ml of culture supernatant plated onto 5% sheep blood agar plates and then incubated overnight at 37°C.

### Proteinase K and heat inactivation treatment of culture supernatants

Bacterial culture supernatants from EHEC were incubated with proteinase K conjugated to agarose beads (10 to 1000 µg/ml, 1h shaking, 37°C) (Sigma Aldrich, Oakville, Ontario, Canada). Culture supernatants incubated with agarose beads and pre-incubated with bovine serum albumin (5% BSA) were used as a negative control. After incubation, agarose beads were removed from the solution by centrifugation (3,000g, 1 min) before incubation with HEp-2 cells. Culture supernatants from EHEC were also heat inactivated by boiling (100°C, 0.5h) and cooled to 37°C before incubation with HEp-2 cells.

### Epithelial cell infection

Infection of HEp-2 cells was performed at a multiplicity of infection (MOI) of 100∶1. Overnight bacterial culture (10mL) was centrifuged (3,000g, 10 min), the supernatant decanted, and bacterial pellets resuspended in 1.0 ml of antibiotic and serum-free MEM. An aliquot of this bacterial suspension (∼1×10^8^ CFU in 0.1 ml) was then used to infect confluent HEp-2 monolayers grown in 6 well plates (∼1×10^6^ cells/well). The cells were infected with either EHEC or EPEC for 6h at 37°C in 5% CO_2_. Cells were then washed with PBS and stimulated with IFNγ (50 ng/ml; 0.5h at 37°C in 5% CO_2_), followed by whole cell protein extracts for immunoblotting. To determine if active bacterial protein synthesis was required to inhibit the phosphorylation of Stat-1, in a subset of experiments epithelial cells and EHEC were incubated with chloramphenicol (100 µg/ml) at 0 to 4h after infectious challenge with (MOI 100∶1, 6h).

### Immunoblotting

Whole cell protein extracts were collected by resuspending epithelial cells in RIPA buffer (1% Nonidet P-40, 0.5% sodium deoxylate, 0.1% sodium dodecyl sulfate [SDS] in PBS) supplemented with 150 mM NaCl, 50 mM sodium fluoride, 1 mM sodium orthovanadate, 20 µg/ml phenylmethylsulfonyl fluoride, 15 µg/ml aprotinin, 2 µg/ml leupeptin, and 2 µg/ml pepstatin A (all from Sigma Aldrich). Aliquots were applied directly onto cells, mixed and left on ice for 0.5h. Re-suspended pellets were centrifuged at 20,000g for 1 min at 4°C. Supernatants were collected and stored at −80°C until further analysis by western blotting.

Immunoblotting was conducted by combining whole cell protein extracts with SDS-PAGE loading buffer in a 1∶1 (v/v) ratio, incubation at 100°C for 3 min, followed by loading into precast 10% polyacrylamide gels (Ready Gel®; BioRad Laboratories, Hercules, CA). Gels were electrophoresed (150 V, 1h at room temperature), followed by protein transfer onto nitrocellulose membranes (BioTrace NT; Pall Corporation, Ann Arbor, MI) (110 V, 1h at 4°C). Membranes were incubated in Odyssey blocking buffer (Mandel Scientific Company Inc., Guelph, Ontario, Canada) for 0.5h at room temperature on a shaker, followed by incubation with primary antibodies (4°C overnight on a shaker). Primary antibodies included rabbit anti-native-Stat-1 (1 in 1,000 dilution; Cell Signaling, Beverly, MA), rabbit anti-phospho-Stat-1 (1 in 1,000 dilution; Cell Signaling), rabbit anti-IRF1 (1 in 2,000 dilution; Sigma-Aldrich), and mouse anti-β-actin (1 in 5,000 dilution; Sigma). Membranes were washed 3 times with PBS+0.1% Tween (5 min per wash) and then incubated with secondary antibodies (1h at RT on a shaker). Secondary antibodies included IRDye 800 goat anti-rabbit IgG (1 in 20,000 dilution; Rockland Immunochemicals, Gilbertsville, PA) and Alexa Fluor® 680 goat anti-mouse IgG (1 in 20,000 dilution; Molecular Probes, Eugene, OR).

Immunoblots were scanned into an infrared imaging system (Odyssey, LI-COR Biosciences, Lincoln, NE), using both the 700 nm and 800 nm channels, at a resolution of 169 µm. Using automated software (LI-COR Biosciences) densitometry was performed to obtain the integrative intensity of positively stained bands. Integrative intensity values for each of the phospho-Stat-1 and native-Stat-1 bands were normalized to the integrative intensity values obtained for the corresponding β-actin bands. Uninfected cells stimulated with IFNγ were used as positive controls, and standardized to 100%. Densitometry values obtained from samples incubated with live bacteria, or sterile culture supernatants, were then calculated as a percentage of the positive uninfected control.

**Column chromatography.** Bacterial culture supernatants were diluted 1 in 3 with 10 mM Tris-HCL buffer (pH 8.0) and applied onto a DEAE Sephacel anion exchange column (Sigma Aldrich, Oakville, Ontario, Canada), and proteins eluted with increasing concentrations of sodium chloride (0 to 1 M). Protein fractions were dialyzed in 10 mM Tris-HCL buffer (pH 8.0) overnight at 4°C, and further separated using a size exclusion column containing Sepharose CL-6B (Sigma-Aldrich). Fractions were screened for activity in inhibiting IFNγ mediated Stat-1-phosphorylation. Active fractions were analyzed by 18% SDS-PAGE followed by silver staining. Candidate proteins were excised and identified using Mass Spectrometry (Advanced Protein Technology Centre at The Hospital for Sick Children, Toronto, Ontario, Canada).

### Isogenic mutant strains

Isogenic *etpD* (1.9Kb), *yodA* (672bp), and *escN* (1.3Kb) mutants were generated from EHEC O157:H7 strain EDL933 using a one-step inactivation technique and primers detailed in **Table 1** [11]. Briefly, EDL933 was transformed with pKD46, and λ-red recombinase expression induced with L-arabinose at 30°C on a shaker until the OD600 reached 0.6. e*tpD* mutants were generated by electroporating linear DNA fragments containing a kanamycin resistance cassette with 5′ and 3′ flanking regions homologous to *etpD* using primers etpDKOP1 and etpDKOP2 on plasmid pKD4 (template plasmid with FLP recognition target sites flanking a 1.6Kb kanamycin resistance gene). *yodA* mutants were generated in the same fashion, except with primers yodAKOP1 and yodAKOP2, and *escN* mutants with primers escNKOP1 and escNKOP2. All mutants generated were verified by PCR.

**Table 1:** Primers employed in this study.

Name	Sequence (5′->3′)
etpDKOP1	GTGTTCACTACAGTAATTTTGGGGGCCATTCCAGGGTGGGGGGCTGAATTGTGTAGGCTGGAGCTGCTTC
etpDKOP2	TTACATCTCCTGCGCATAAAACGCAGCAATCGCCGCTTTCACCTTCCGGACATATGAATATCCTCCTTAG
escNKOP1	ATGATTTCAGAGCATGATTCTGTATTGGAAAAATACCCACGTGTAGGCTGGAGCTGCTTC
escNKOP2	GGCAACCACTTTGAATAGGCTTTCAATCGTTTTTTCGTAACATATGAATATCCTCCTTAG
YodAKOP1	TTGGCGATTCGTCTTCACAAACTGGCTGTTGCTTTAGGTGTCTTTATTGTGTGTAGGCTGGAGCTGCTTC
YodAKOP2	TCAATGAGACATCATTTCCTCGACCACTTCTTCGCTACTCAACTGATATGCATATGAATATCCTCCTTAG
yodA-C'P1	GAGGAATAATAAATGACTCTGGAGGAAACTGTTTTGG
yodA-C'P2	ATGAGACATCATTTCCTCGACCAC

### EHEC pO157 plasmid removal

The pO157 plasmid was cured from EHEC using the pCURE2 kit from Plasgene (Plasgene, Birmingham UK) [12]. Briefly, *Escherichia coli* O157:H7 strain EDL933 was transformed with the pCURE2 plasmid which displaces the pO157 plasmid, and transformants were selected for resistance on LB kanamycin plates (50 µg/ml). Transformants were then counter-selected for sucrose sensitivity (*sacB*) by incubation on LB plates supplemented with 5% sucrose, and colonies recovered were verified for their loss of the pO157 and pCURE2 plasmids by PCR.

### YodA cloning, over-expression and purification

To over-express and purify the YodA protein (24.6kDa), the *yodA* gene was cloned into the pBAD-TOPO TA cloning kit (Invitrogen) under control of the arabinose promoter. Briefly, the *yodA* gene was PCR amplified using primers yodA-C'P1 and yodA-C'P2, cloned upstream of a 6x-His tag to generate pBAD-yodA-His, and verified using PCR and DNA sequencing (Centre for Applied Genomics, Hospital for Sick Children, Toronto, Ontario, Canada). YodA over-expression was induced in *Escherichia coli* DH5α with 0.2% arabinose and purified using nickel column chromatography employing a Qiagen His-tagged purification kit (Qiagen, Toronto).

### Statistics

Results are expressed as means, ± standard error (SE). Levels of Stat-1 tyrosine phosphorylation were compared by using one-way analysis of variance (ANOVA), with Tukey's multiple comparison test. Analyses were performed using Prism4 (GraphPad, San Diego, California, USA). Differences of p<0.05 were considered significant.

## Results

### EHEC, but not EPEC, inhibits IFNγ mediated Stat-1 tyrosine phosphorylation via secreted factors

**Figure 1:**
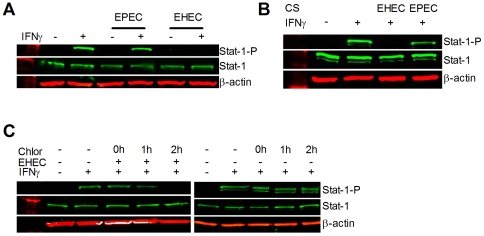
EHEC, but not EPEC, inhibits IFNγ mediated Stat-1 tyrosine phosphorylation. Whole-cell protein extracts from HEp-2 cells analyzed by immunoblotting showed that (**A**) IFNγ mediated (50ng/ml, 0.5h) Stat-1 tyrosine phosphorylation is suppressed by enterohemorrhagic *Escherichia coli* O157:H7, strain EDL933 (EHEC), but not by enteropathogenic *Escherichia coli* O127:H6, strain E2348/69 (EPEC) (MOI of 100∶1; 6h). (**B**) Incubation with sterile culture supernatants (6h) from EHEC, but not EPEC, suppressed IFNγ-mediated tyrosine phosphorylation of Stat-1. (**C**) Incubation with chloramphenicol (100 µg/ml) up to 1h after infection with EHEC (MOI 100∶1; 6h) prevented EHEC suppression of IFNγ mediated Stat-1 tyrosine phosphorylation.

To demonstrate that EHEC, but not EPEC, was able to subvert the IFNγ pathway, we assessed the tyrosine phosphorylation state of Stat-1 in cell protein extracts. In un-stimulated epithelia, Stat-1 is normally not tyrosine phosphorylated, but becomes activated in response to IFNγ stimulation [5], [9]. Infection with either pathogen does not affect native Stat-1 expression (**Figure 1A**), however EHEC prevented Stat-1 tyrosine phosphorylation in response to IFNγ stimulation (**Figure 1A**). By contrast, EPEC did not inhibit IFNγ mediated Stat-1 tyrosine phosphorylation [9,13-Submitted], indicating that the ability to subvert IFNγ signaling is a specific ability of EHEC, and not all pathogenic *E. coli*.

Incubation of HEp-2 cells with sterile culture supernatants showed that EHEC, but not EPEC, culture supernatants are able to inhibit IFNγ mediated Stat-1 tyrosine phosphorylation, with no affect on native Stat-1 levels (**Figure 1B**) [13-Submitted]. The addition of chloramphenicol within 1h of EHEC infection prevented bacterial inhibition of Stat-1 tyrosine phosphorylation in response to IFNγ (**Figure 1C**), suggesting that subversion of IFNγ mediated Stat-1 tyrosine phosphorylation is time dependent, and requires new and active bacterial protein synthesis [13-Submitted].

### Secreted inhibitory factor(s) have protein-like qualities

**Figure 2:**
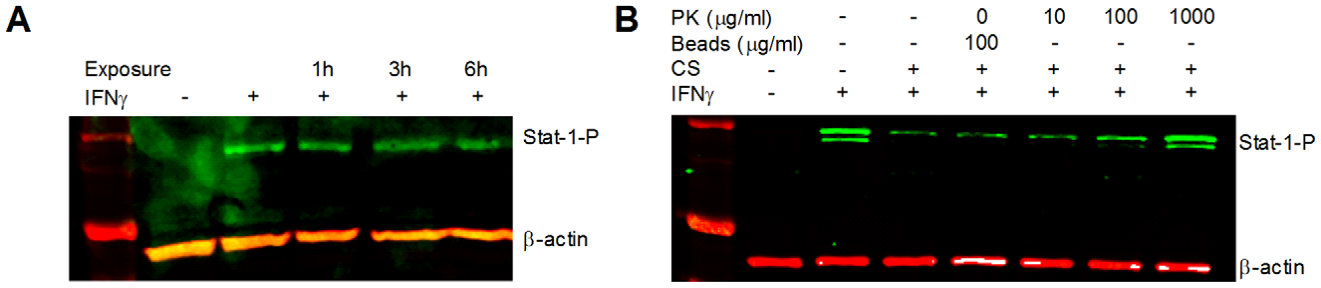
Secreted factor(s) have protein qualities. Whole cell protein extracts from HEp-2 cells analyzed by immunoblotting showed that (**A**) Incubation with heat treated (100°C, 0.5h) EHEC CS did not suppress IFNγ mediated Stat-1 tyrosine phosphorylation, and (**B**) incubation with EHEC CS (6h) pre-treated with proteinase K conjugated to agarose beads (0 – 1,000mg/ml, 37°C, 1h) did not suppress IFNγ induced tyrosine phosphorylation of Stat-1 (n = 3, one-way ANOVA, * p<0.05).

To verify that the inhibitory effects of EHEC were not due to heat resistant factors, such as lipopolysaccharide [14], [15], culture supernatants were incubated at 100°C for 30 min and then assessed for the ability to inhibit Stat-1 tyrosine phosphorylation. As observed previously in our laboratory [13-Submitted], boiled culture supernatants did not inhibit Stat-1 tyrosine phosphorylation, even after 6h of incubation with tissue culture epithelial cells (**Figure 2A**).

To confirm that the inhibitory factor(s) were protein in nature, bacterial culture supernatants were treated with proteinase K, and then tested for their ability to inhibit Stat-1 tyrosine phosphorylation. Culture supernatants treated with agarose beads as a negative control still blocked signaling responses to IFNγ, while those treated with increasing concentrations of proteinase K progressively lost the ability to subvert IFNγ mediated Stat-1 tyrosine phosphorylation (**Figure 2B**) [13-Submitted].

### EHEC secreted inhibitory factor identified as YodA

**Figure 3:**
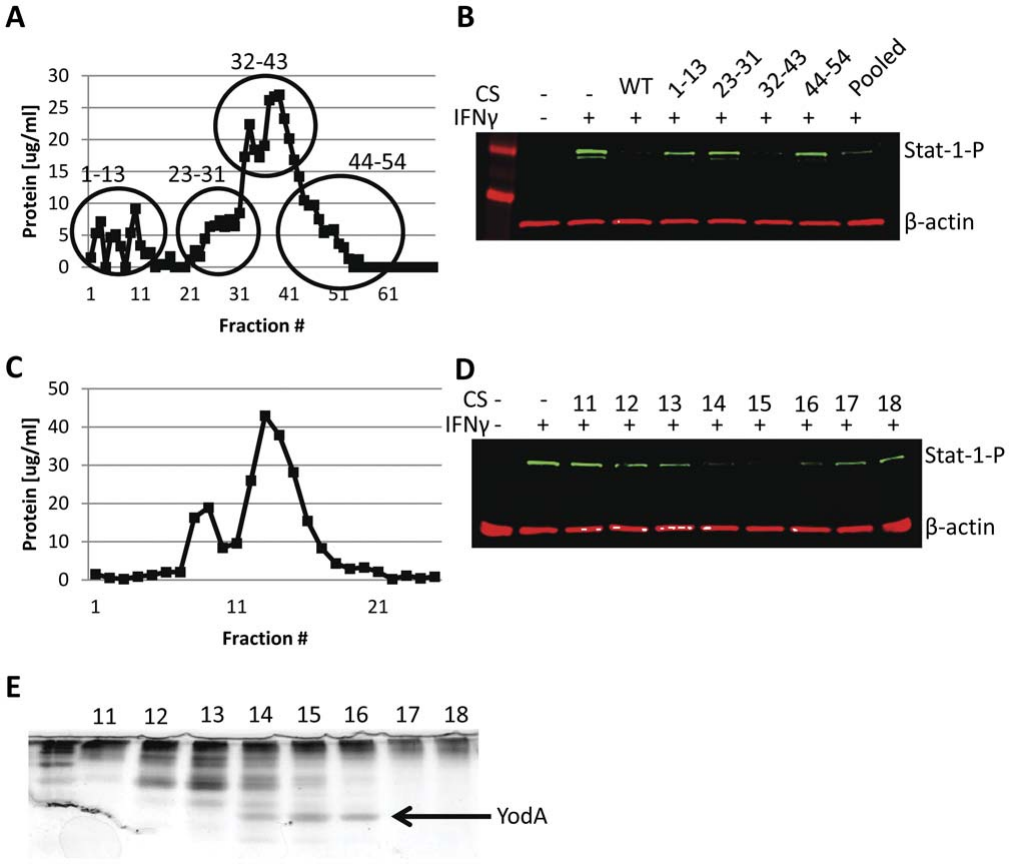
EHEC secreted inhibitory factor identified as YodA. A Bradford assay was performed on EHEC culture supernatants separated by anion exchange (**A**). A 1.2cm by 50cm column containing DEAE Sephacel that had been mixed with 0.5L of culture supernatant was initially rinsed with low salt buffer prior to being eluted with a 0 to 1M gradient of NaCl. Three mL fractions were collected and protein content was determined by Bradford assay. Fraction 60 represents a NaCl concentration of ∼200mM and no further protein elution at higher salt concentrations was observed. Fractions of interest were pooled and dialyzed before incubation with HEp-2 cells, and tested for their ability to inhibit IFNγ mediated Stat-1 tyrosine phosphorylation (**B**). Pooled fractions 32–43 from the DEAE Sephacel column were concentrated by membrane filtration prior to being subjected to size exclusion chromatography using a 1.2cm by 50cm Sepharose CL-6B column equilibrated with 10 mM Tris-HCL buffer (pH 8.0). Three mL fractions were collected and the protein concentration present was determined by Bradford assay (**C**). Select fractions from size exclusion chromatography were tested for their ability to inhibit IFNγ mediated Stat-1 tyrosine phosphorylation (**D**). Fractions of interest were separated using 18% SDS-PAGE and stained with silver (**E**).

**Figure 4:**
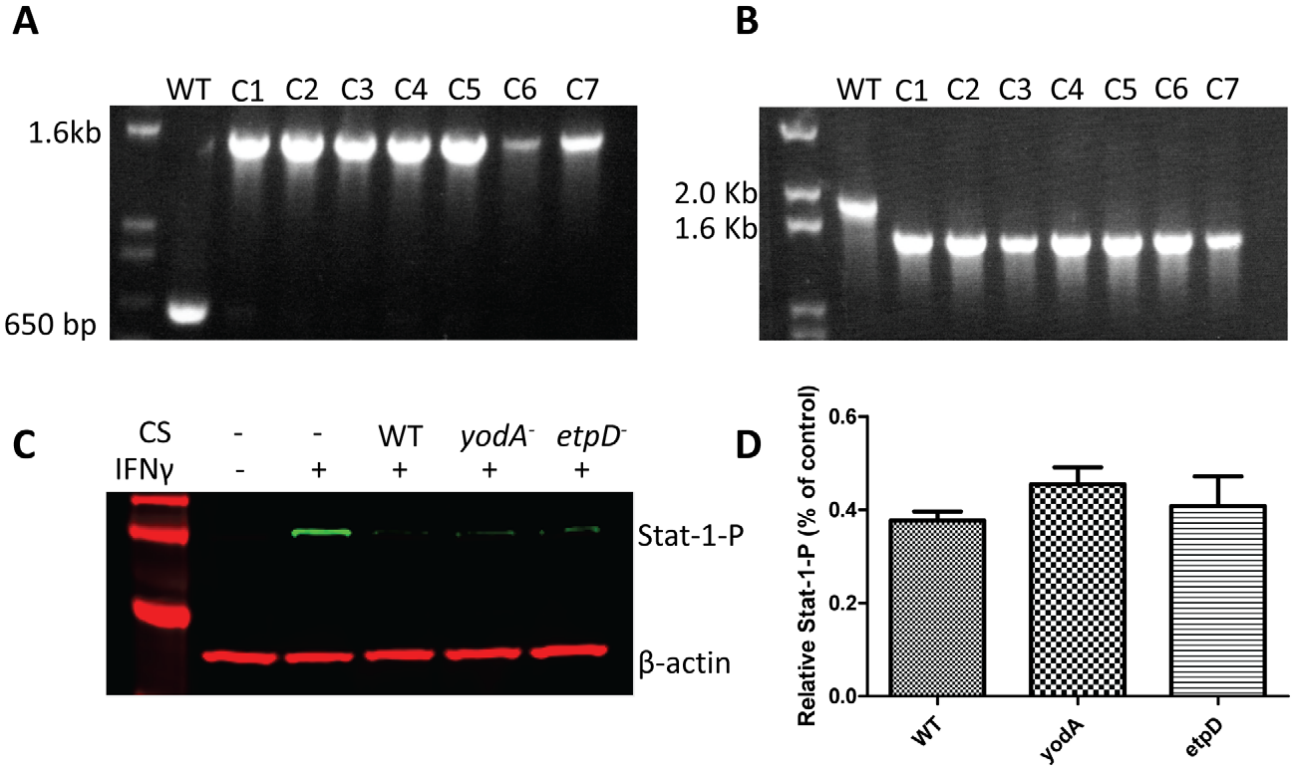
Isogenic *yodA* and *etpD* mutants inhibit Stat-1 tyrosine phosphorylation at levels comparable tos wild-type. Isogenic *yodA* and *etpD* mutants were verified by PCR (panels **A** and **B**, respectively). Whole-cell protein extracts from HEp-2 cells were incubated with sterile culture supernatants from WT, *yodA* and *etpD* mutants and then tested for their ability to inhibit IFNγ mediated Stat-1 tyrosine phosphorylation; representative immunoblot (**C**) and repeated experiments analyzed by densitometry (n = 3) (**D**).

In order to identify the secreted inhibitory factor(s), we separated the protein contents of EHEC culture supernatants based on charge density differences using anion exchange chromatography. After an initial loading and low salt rinsing step, bound material was eluted using a linear salt gradient, and fractions were collected at regular intervals and analyzed for their protein concentration using the Bradford assay (**Figure 3A**). Fractions from distinct regions of the anion exchange profile were pooled into 4 groups, dialyzed, and incubated with HEp-2 cells. Only fractions 32–43 inhibited IFNγ mediated Stat-1 tyrosine phosphorylation (**Figure 3B**). In order to further identify the inhibitory factor(s), fractions 32-43 from the DEAE column were pooled and separated using size exclusion chromatography to separate the proteins based on molecular mass. Fractions from the size exclusion column were then retested for protein concentration using the Bradford assay (**Figure 3C**), and tested for their ability to inhibit IFNγ mediated Stat-1 tyrosine phosphorylation (**Figure 3D**). According to western blotting, fractions 13–16 showed inhibitory qualities, which appeared to correspond to the presence of a single band present in fractions 14–16 when separated by SDS-PAGE (**Figure 3E**). The band was excised, digested by trypsin, and identified as YodA by standard methods of peptide fingerprint analysis using mass spectrometry (Advanced Protein Technology Centre at The Hospital for Sick Children, Toronto, Ontario, Canada). YodA is a metal binding protein of unknown function [16], [17]. To determine if YodA played a role in Stat-1 suppression, isogenic *yodA* mutants in *E. coli* O157:H7 strain EDL933 were created using the λ-red knockout system [11] along with the *E. coli* Type-2 Secretion system (*etpD*) which is required for YodA excretion [18]. Both isogenic mutants were verified by PCR (**Figure 4A, B**), and then assessed for their ability to inhibit Stat-1-phosphorylation (**Figure 4C, D**).

**Figure 5:**
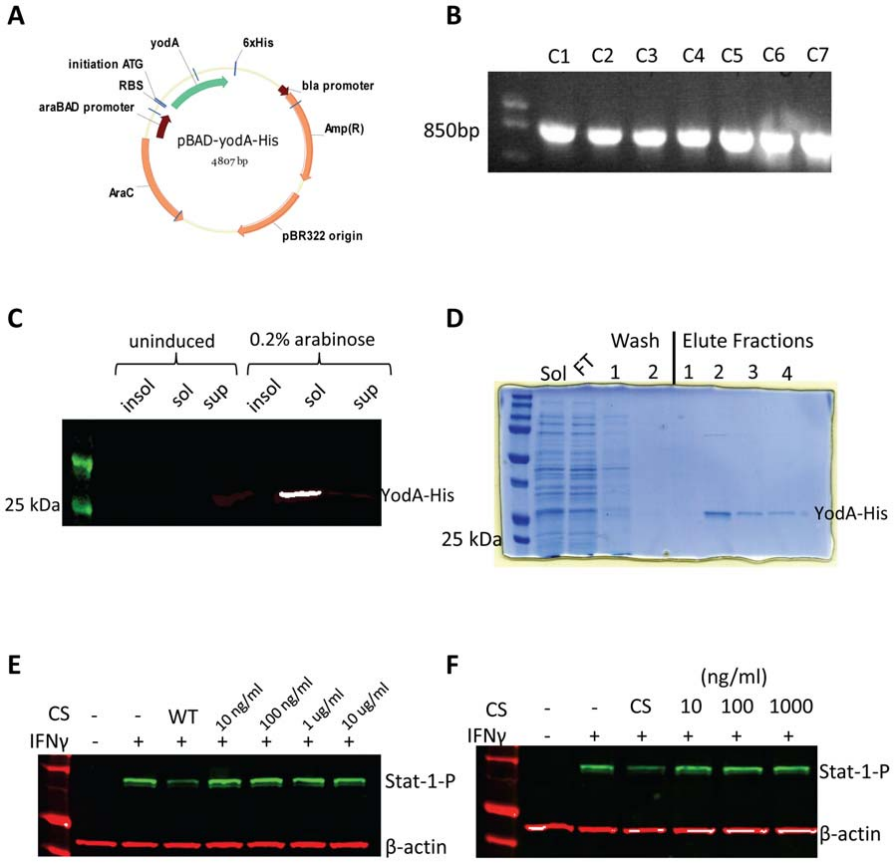
Purified YodA failed to inhibit Stat-1 tyrosine phosphorylation as well as wild-type culture supernatant. EHEC *yodA* gene was cloned into the pBAD protein expression vector (**A**) and verified by PCR (**B**). Over-expressed YodA-His was detected in the soluble fraction, but not in the insoluble or supernatant fraction, by western blot (**C**) and purified using nickel column chromatography (**D**). Varying concentrations of eluted and dialyzed YodA-His were tested for the ability to inhibit IFNγ mediated Stat-1 tyrosine phosphorylation (panels **E** and **F**, respectively).

Contrary to expectations, both isogenic mutants inhibited Stat-1-phosphorylation at levels comparable to the wild-type EHEC strain, indicating that neither *yodA* nor *etpD* was involved in EHEC subversion of IFNγ/Stat-1 signaling. To verify the mutation experiments, the *yodA* gene was cloned into the pBAD-TOPO protein expression system with a C-terminal histidine tag (**Figure 5A**), and verified by PCR (**Figure 5B**) and sequencing. Protein over-expression and solubility was verified using western blotting (**Figure 5C**) and then purified by nickel column chromatography (**Figure 5D**). Varying concentrations of the eluted and dialyzed YodA-His protein (**Figure 5E, F**, respectively) were then tested directly on HEp-2 cells; however, purified YodA did not inhibit IFNγ mediated Stat-1 tyrosine phosphorylation.

## Discussion

In the present study, we have shown that EHEC, but not EPEC, subverts the IFNγ signaling pathway at the level of Stat-1 tyrosine phosphorylation. Direct contact of the non-invasive enteric pathogen with host cells is not required to mediate this effect, as extracellular bacterial culture medium was able to produce the effect associated with the subversion factor in the absence of intact bacteria. Through a series of biochemical tests, we establish in this report that the secreted bacterial factor has properties consistent with it being a protein (Figure 2), but it is not secreted via the type two secretion protein export system.

Recent studies have found that subversion of innate immune pathways is a theme common to multiple pathogens, illustrating the important role of the IFNγ signal transduction pathway in combating microbial infections [3]. For example, viruses of the *Paramyxoviridae* family subvert the IFNγ pathway by degrading intracellular Stat-1 [19], while the parasite *Leishmania donovani* prevents tyrosine-phosphorylation of Stat-1 [20]. The ability of EHEC O157:H7 to suppress the IFNγ pathway could promote its ability to colonize the gut of the infected host by reducing immune surveillance [3].

EHEC and EPEC both contain an array of virulence factors that aid in infection, of which the LEE (Locus of Enterocyte Effacement) pathogenicity island encoded type three secretion system (T3SS) is common to both; allowing the bacterium to adhere intimately onto host cells. The LEE's of EHEC and EPEC were acquired through horizontal gene transfer and provide these non-invasive pathogens the ability to deliver effector proteins directly into host cells [21]. These effectors allow the bacterium to subvert host cytoskeleton processes, destroy brush border microvilli, and cause rearrangements of F-actin resulting in attaching and effacing (A/E) lesions on epithelial cell surfaces [22], [23], [24].

**Figure 6:**
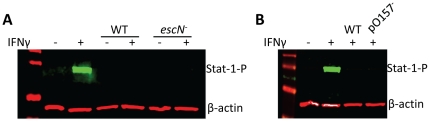
EHEC LEE and pO157 are not involved in the suppression of the IFNγ signaling pathway. Isogenic *escN*, and pO157 cured mutants were tested for their ability to inhibit IFNγ mediated Stat-1 tyrosine phosphorylation (**A** and **B**, respectively).

Studies on the T3SS and its protein effectors have shown that many of these bacterial-derived proteins have redundant and overlapping functions, each of which appears to have multiple roles in subverting eukaryotic cellular processes, and thereby aid in disease pathogenesis [25]. Despite both pathogens having a LEE, they are not identical, and many of the effectors, while having similar effects on host cells, can vary between the two enteric pathogens [26], [27]. However, the EHEC LEE is not involved in subverting the IFNγ signaling pathway, as demonstrated in this report by showing that an isogenic *escN* mutant suppresses Stat-1 tyrosine phosphorylation comparable to the parental wild-type strain (**Figure 6A**).

EHEC also contains other virulence factors distinct from EPEC, which gives this enteric pathogen the ability to inhibit Stat-1 tyrosine phosphorylation. For instance, EHEC contains phage encoded Shiga toxins, which are responsible for causing hemorrhagic colitis and hemolytic-uremic syndrome [28], [29]. EHEC also harbors the pO157 virulence plasmid that encodes a metalloprotease (*stcE*) [30], a serine protease (*espP*) [31], a hemolysin (*ehxA*) [32], a catalase-peroxidase (*katP*) [33], a putative adhesion (*toxB*) [34], as well as 22 other open reading frames that have no strong similarity with any known proteins [35]. However, the EHEC pO157 plasmid is also not involved in subverting the IFNγ signal transduction pathway, because a pO157 plasmid cured mutant suppressed Stat-1 tyrosine phosphorylation comparable to the parental wild-type strain (**Figure 6B**).

The expression of the YodA protein in bacterial supernatants leads to its co-purification with the Stat-1-P inhibitory activity that was present in the sample. Further, the relatively small size of the protein, ∼25kD, was consistent with the observed behavior of the inhibitory activity when supernatant was passed through filters with defined molecular cut–offs. YodA therefore appeared to match the characteristics of the inhibitor molecule, however our inability to detect Stat-1-P suppression in the presence of overly expressed YodA (**Figure 5C–D**) suggests that this protein is either not the inhibitor, or alternatively it is dysfunctional in the form in which it is expressed. It's recovery as a small molecule on the size exclusion column suggests that it does not natively pair with another protein or entity for activity, however the resolution of the DEAE and the Sepharose CL-6B columns may not be sufficient to detect if specific post-translational modifications are required to create the activate form of the YodA protein. Nonetheless, the inability of the purified YodA protein to suppress Stat-1 tyrosine phosphorylation, in addition to the *yodA* mutant's preserved ability to suppress Stat-1 tyrosine phosphorylation, indicates that EHEC likely utilizes a different protein factor to suppress IFNγ activation.

EHEC undoubtedly contains other virulence factors which are distinct from EPEC, but which are currently uncharacterized. Recent studies have shown that EHEC, strain EDL933 (5.62Mb; 5,312 genes) is roughly 11% larger than EPEC strain E2348/69 (5.07Mb; 4,554 genes) [36]. Further research focusing on the differences between the EHEC and EPEC genomes will elucidate the EHEC-derived protein factor that is responsible for the subversion of the IFNγ signaling pathway. Identification of this inhibitory factor(s) could potentially be used to restore host cell signaling responses following EHEC infection and, thereby, reduce the severity and spread of this infectious agent.
